# 

**Published:** 1836-10-01

**Authors:** 


### BIBLIOGRAPHICAL RECORD;

OR,

*Works received for Review since last Quarter.*

1. Thoughts on Physical Education, and the true Mode of improving the Condition of Man, &c. By CHARLES CALDWELL, M.D.; with Notes, by ROBERT COX, and a recommendatory Preface by GEORGE COMBE. Duodecimo, pp. 190. Edinburgh, 1836.

---

2. A Dictionary of Terms Employed by the French in Anatomy, &c. &c. &c. By SHIRLEY PALMER, M.D. Part the Second. Birmingham. June, 1836.

---

3. An Engraving of John Hunter, executed by W. O. GELLER, from a painting by Sir Joshua Reynolds, in the College of Surgeons.

☞ *This is an admirable engraving, and published on very moderate terms.*

---

4. CATARACT: a familiar description of its symptoms, nature, and ordinary treatment, &c. &c. By JOHN STEVENSON, Esq. Second Edition, carefully revised and enlarged. Small Octavo, pp. 139. Highley, June, 1836.

---

5. A Series of Anatomical Plates, &c. &c. By Dr. QUAIN. Divis. 2. Arteries x. xi. July, 1836.

☞ *The delineation of the arteries continues to occupy these Plates. They sustain their character for beauty and utility—no trifling praise. We would suggest the avoidance of deep shading as much as possible. Bold, broad outline is most effective in diagrams.*

---

6. A History of British Quadrupeds. By THOMAS BELL, F.R.S. F.L.S. Lecturer on Comparative Anatomy at Guy's Hospital. Part 1. Price 2s. 6d. Illustrated by a wood-cut of each species, and numerous vignettes. London. Sherwood and Co. July, 1836.

☞ *This part is occupied with the genus* VESPERTILIO. *The vignettes are executed beautifully.*

7. Suggestions for obtaining the best Medical Advice at the least possible Expense. Addressed to Persons of limited Income, but respectable Station in Society. Octavo, pp. 8.

☞ *The object appears to be the formation of a kind of joint-stock company, for securing doctors and drugs of good quality (and fairly paid) by annual contributions. It is, after all, a benefit-society. We doubt its practicability—or, at all events, its success. We have made some observations on the subject in the present Number.*

---

8. St. Thomas's Hospital Reports. By JOHN F. SOUTH, Assistant-Surgeon. No. 4, June, 1836, price 3s. Sherwood and Co.

---

9. Elements of General Anatomy; translated from the last Edition of P. A. Beclard, Professor of Anatomy to the Faculty of Medicine in Paris, with Notes and Corrections, by ROBT. KNOX, M.D. F.R.S. E. Lecturer on Anatomy, &c. Edinb. Octavo, pp. 396. Maclachlan and Stewart, 1836.

---

10. Sixteenth Report from the Dundee Lunatic Asylum, ending May 31st, 1836.

---

11. A History of British Quadrupeds. By THOMAS BELL, FRS, &c. Part II., illustrated with Wood-cuts. August, 1836.

☞ *This Part concludes the subject of Bats, and includes the Hedgehog and Mole. The work well deserves the patronage of the public.*

---

12. Researches in Medicine and Medical Jurisprudence. By JOHN B. BECK, M.D. Second Edition, New York, 1835.

☞ *These essays are five in number, all of which have previously appeared in print:—1, Infanticide—2, Acute Laryngitis—3, Non-contagion of Yellow Fever—4, Onychia Maligna—5, Ulceration and Perforation of the Stomach. Some of these we shall notice.*

13. The Practical Anatomy and Elementary Physiology of the Nervous System; designed for the Use of Students in the Dissecting-Room. By JAMES CLARK, Demonstrator of Anatomy in St. Thomas's Hospital. Octavo, pp. 357. Longman and Co. 1836.

---

14. The Proofs of Infanticide considered; including Dr. Hunter's Tract on Child-Murder, with illustrative Notes, and a Summary of the present State of Medico-legal Knowledge on that Subject. By WILLIAM CUMMIN, M.D. Lecturer on Forensic Medicine, &c. 12mo, pp. 95. Longman and Co. Aug. 1836.

---

15. Introductory Lecture delivered to the Mathematical Class at the Royal School of Medicine and Surgery at Birmingham. By the Reverend W. MORELL LAWSON, M.A.

☞ *We are glad to find that the exact sciences are beginning to be blended with the artes conjecturales. Birmingham has here given a lesson to London.*

---

16. Inaugural Dissertation on the Utility of Dispensaries, &c. By JOHN NELSON, M.D. Octavo, pp. 32. 1836.

---

17. A few Hints on the Principle of Provident or Self-supporting Dispensaries, and its Adaptation to the Medical Relief of the Poor. By CHARLES WILKINSON, M.D. 12mo, sewed, pp. 47. 1836.

---

18. Address to the Governors and Subscribers of the Charing-Cross Hospital, on some extraordinary Proceedings that have lately taken place at that Institution, &c. By T. J. PETTIGREW, F.R.S. &c.

"Non nostrum inter vos tantas componere lites."

---

19. Researches into the Physical History of Mankind. By JAS. COWLEY PRICHARD, M.D. F.R.S. &c. 3d Edit. Vol. I. Aug. 1836.

☞ *This edition is almost entirely re-written, and greatly enriched by modern additions to our knowledge.*

---

20. Principles of Pathology, and Practice of Physic. By JOHN MACKINTOSH, M.D. Lecturer &c. *Fourth Edition*, 2 Vols. 8vo, pp. 524—546. Sept. 1836.

☞ *These Volumes came too late for notice this quarter (18th Sept.); but we shall introduce a short article in our next Number, especially on the pathology of cholera.*

---

21. A very Comprehensive Index to Dr. Davis's Obstetric Medicine. 4to, stitched, 1836.

☞ *It is very comprehensive.*

---

22. Manuel de Médecine Operatoire, fondée sur l'Anatomie Normale et l'Anatomie Pathologique. Par S. F. MALGAIGNE, M.D. &c. Second Edit. revised, corrected, and considerably enlarged. Baillière, Regent-street, 1836.

☞ *This edition is very considerably improved, and the author appears to have omitted no labour or pains, "pour maintenir l'ouvrage au niveau de la science." We can confidently recommend this manual.*

---

23. A concise Anatomical Description of the Arteries of the Human Body, together with full Directions for cutting down upon and securing the several Arterial Trunks; for the Use of Students in Anatomy. By P. BENNETT LUCAS, M.R.C.S. &c. 12mo, pp. 116. London, 1836.

☞ *This is really what it purports to be, a concise description of the arteries of the human body. And it is as clear as it is concise. Altogether, it is a very excellent little manual for the student to dissect by.*

---

24. The Report of a Committee on the new Poor-Law Act, appointed by the Provincial Medical and Surgical Association, &c. Octavo, stitched, pp. 38. Worcester, 1836.

---

25. *Analytical Anatomy.*—The Circulation of the Blood, as found in the Human Fœtus, and compared with that in the Five Classes of Vertebral Animals. By G. J. MARTIN SAINT-AINGE, D.M.P. Translated from the French, by T. W. JONES, D.M.P. In Folio.

---

*Closed on the 19th September.*

### INDEX.

A

Absorption of chyle .............. 111
Accidental uterine hæmorrhage...... 58
Act respecting medical witnesses.... 568
Acute hydrocephalus .............. 75
Acute articular rheumatism, Bouillaud on .................... 82
Acute pericarditis, Dr. Roots on ... 455
Additional medical evidence ........ 568
Advantages of new remedies........ 158
Affections of the heart, digitalis in .. 229
Agues, endermic use of quinine in .. 228
Air-pump, Sir J. Murray on the .... 346
Albinism and melanism............. 234
Alexander on puerperal fever, &c..... 72
Alibert on cheloid tumor of the skin 231
Amputation of the hand by machinery 561
Amputation, hæmorrhage after...... 573
Anatomical preparations, closure of.. 136
Anatomical plates, by Dr. Quain.... 207
Anatomist's instructor, by Mr. Knox 408
Anatomy, practical, of the nerves, &c. 205
Anatomy, on the study of.......... 483
Andral's clinique médicale....... .. 207
Aneurism of the ascending aorta .... 447
Aneurism, mode of applying the ligature for .................. 561
Animals, nutrition of ............ 102
Anomalous hysterical affection ...... 259
Antidote to arsenic................ 518
Antimony in delirium of fevers, Graves on .......................... 437
Arsenic, antidote to .............. 518
Arteriæ helicinæ corporis cavernosi .. 189
Articular rheumatism, inflammatory nature of...................... 86
Articular rheumatism, specific for .. 88
Ashwell on midwifery ............ 38
Atrophy, nature and causes of...... 120
Atrophy, first form of ........... 122
Atrophy, state of liver in.......... 125
Atrophy, second form of .......... 126
Atrophy, remarkable case of........ 126
Atrophy, third form of ........... 126
Atrophy of the gall-bladder ........ 127
Auscultation .................... 50
Auscultation .................... 79
Autumnal fevers, &c., Nieuwenhuys on the ........................ 131

B

Barlow on ovarian tumors.......... 360
Barlow's, Mr. G. H. reclamation . .. 586
Belfast fever hospital.............. 547
Beriberi ........................ 342
Bile, composition of the .......... 109
Birmingham infirmary, report of the.. 528
Blackening of the skin, case of...... 237
Bladder, vaginal hernia of the ...... 164
Bladder, stone in the.............. 537
Bleeding in the cold stage of intermittents ........................ 278
Blizard, Sir W. memoir of ........ 280
Blockley hospital.................. 562
Bones, method of cleaning of ...... 418
Bones of the fore-arm, ununited fracture of........................ 430
Bones of the leg, ununited fracture of 431
Bostock on mercury in onychia maligna 474
Bouillaud on acute articular rheumatism ......................... 82
Bouillaud's treatment of rheumatism 90
Brain, preservation of the.......... 417
Breech presentations .............. 52
Breschet's operation for varicocele .. 463
Bright on renal disease ............ 23
Bringing up of children by hand .... 18
Burning of the feet .............. 341
Burning of the feet, symptoms of.... 343

C

Cancer, use of creosote in.......... 71
Cantharides, poisoning by ......... 461
Carswell on the elementary forms of disease......................... 120
Case of recovery from uterine hæmorrhage ........................ 57
Case of puerperal convulsions . .... 63
Case of crying of the child in utero.. 66
Case of burning of the feet ........ 342
Case of neuralgia of the uterus...... 355
Case of deformity of the pelvis...... 143
Case of blackening of the skin ...... 237
Case of twins .................... 495
Cases of laryngismus stridulus ...... 5
Cases of renal disease.............. 24
Cases of albuminous urine, exhibition of mercury in ................... 33
Cases of the effects of secale cornutum 349
Cases of disease of the kidney, by H. J. Johnson ...................... 193
Causes of laryngismus stridulus .... 10
Celsus by Alexander Lee............ 444
Cephalopoda, ink-bag of the........ 203
Cervix femoris, mode of treating .... 260
Changes of urine in disease ........ 208
Charter of the University of London.. 583
Chavasse on secale cornutum ...... 349
Cheloid tumor of the skin, Alibert on 231
Chemical view of the process of digestion ........................... 110
Chemical theory of respiration ...... 112
Chemistry, meteorology, &c. by Dr. Prout ......................... 95

Chemistry, reflections on .......... 115
Chloride of zinc, Ure on the........ 190
Chloride of zinc, modes of preparing the .......................... 190
Chloride of zinc, mode of using the.. 192
Chlorine ........................ 108
Chorea, fatal case of .............. 451
Chronic dysentery ................ 246
Chronic laryngitis, Dr. Roots on .... 449
Chronic laryngitis, clinical remarks on 450
Chyle, absorption of .............. 111
Cicatrix, properties of a............ 203
Cirrhosis ........................ 125
Clavicle, dislocation of the ........ 246
Clinical medicine, Dr. Latham on.... 75
Clinique medicale, by Dr. Andral.... 207
Closure of moist anatomical preparations ........................ 136
Cochlea, on the use of the.......... 177
Cock's practical anatomy of the nerves, &c. ........................ 205
Coincidence of endocarditis and pericarditis ........................ 515
Colchicum, use of, in tetanus ...... 161
Coley on hydrometra .............. 357
Collins on midwifery .............. 38
Common causes of death in infants.. 527
Comparative frequency of the eruptions ........................ 403
Comparative mortality of males and females ........................ 172
Comparative mortality of the married and unmarried ................ 175
Compendium of the ligaments ...... 206
Compound fracture of the skull .... 167
Congenital occlusion of the vagina .. 522
Congenitally imperforate vagina, case of ........................ 526
Cormack on the properties of creosote 69
Coulson on tight lacing............ 460
Coulson on deformities of the chest.. 477
Cramp .......................... 117
Cranium and chest, osteology of the.. 490
Creosote, treatise on the properties of 69
Creosote, most important property of 70
Creosote, physiological effects of .... 70
Creosote, a remedy for tooth-ache .. 71
Creosote, useful in cancer.......... 71
Cretinism ...................... 118
Crotale ........................ 118
Crypt .......................... 119
Crystals on the peritoneum, Harrison on ........................ 180
Cummin, Dr: on infanticide ........ 322
Cull, Mr. on stammering .......... 319
Cutler's surgeon's practical guide.... 206
Cure of an obstinate ozœna ........ 514
Cyclopædia of anatomy by Todd .... 202

D

Danger, &c. of quackery............ 149
Davis on obstetric medicine ........ 36
Death, age at which it occurs ...... 567
Decapitation, Rigby on the philosophy of ........................ 151
Deformities of the chest, Mr. Coulson on ........................ 477
Deformity of the pelvis, case of, by Knowles ........................ 143
Description of laryngismus stridulus 4
Description of hydrometra.......... 358
Description of ecthyma ............ 392
Detection of sulphate of quinine in urine ........................ 215
Dewees on the exhibition of secale cornutum ........................ 351
Dictionary of terms, by Dr. Palmer.. 117
Diffused tuberculation ............ 256
Digestion, chemical view of the process of ........................ 110
Digitalis in affections of the heart.... 229
Disease of the kidney, curious case of 195
Disease, on the changes of urine in .. 208
Disease of the female pudendum .... 352
Diseased bones, preparation of ...... 424
Diseased lung, preservation of ...... 427
Diseases of the joints.............. 248
Dislocation of the spine, recovery from 185
Dislocation of the clavicle.......... 246
Doctors, mortality of .............. 174
Douglas on phlebitis .............. 263
Dropsy, with ovarian tumors ...... 473
Dublin Medical Journal............ 279
Duchatelet on prostitution in Paris .. 333
Dyspepsia, appearance of urine in.... 210

E

Eastern Prov. Med. Association...... 286
Ecthyma, description of............ 392
Ecthyma, concomitants of.......... 393
Edmonds's statistics of the London Hospital ........................ 555
Elementary forms of disease, Carswell on ........................ 120
Embryotomy, when to be resorted to 55
Emerson on infantile mortality in Philadelphia ........................ 169
Endocarditis .................... 83
Endocarditis and pericarditis, coincidence of ........................ 515
Enlargement of the ovary.......... 41
Enuresis ........................ 444
Epidemic cholera, Parkin on........ 480
Eruptions, comparative frequency of the ........................ 403
Europeans in India, rheumatism of .. 344
Exanthema roseolum.............. 375
Exanthema puniceum .............. 376
Excision of the cyst of a strangulated umbilical hernia ................ 182
Exhalation from the skin .......... 236
Extraction of a pin from the female bladder ........................ 452

F

Factories, statistics of.............. 175

Fatal case of chorea, Dr. Johnson's .. 451

Female pudendum, disease of the . .. 352
Femur, ununited fracture of the .... 429
Feet, burning of the .... ........ 341
Feet, burning of the, case of... .... 342
Fever, statistics of ................ 547
Fever, predisposing causes of ...... 550
Fever, influence of trade as a cause of 551
Fever, females more susceptible to than males ...................... 552
Fever, average duration of.......... 552
Flooding ........................ 56
Formulary of hospitals by Dr. Ryan.. 199
Fracture, cases of ununited ........ 428
Fracture of the patella, cases of...... 561
Fractured leg, maltreatment of a .... 181
Fright, effects of..... ............. 574
Fungoid tumor followed by melanosis, case of......................... 368
Furlong on the yellow fever at Antigua 289

G

Gall-bladder, atrophy of the ........ 127
Gangrene of the lungs, Guislan on .. 509
Gangrene of the lungs, case of ...... 511
Gerhard's reports on pneumothorax.. 562
German contributions to medicine, by Dr. Thacker..................... 163
Glanders in the human subject, cases of ............................ 516
Glanders-infection in the human subject .......................... 517
Gold-pointed lancet .............. 587
Graves on antimony in delirium of fevers ....................... 437
Green on mesenteric disease ........ 282
Guislan on gangrene of the lungs in the insane .......... ........ 509
Gun-shot wound of the face and neck 441

H

Hæmorrhage after amputation ...... 573
Hall on the nervous system...... 92, 293
Hallowell on rupture of the heart.... 152
Hamilton on midwifery............. 38
Hamilton's case of periostitis of orbit, 176
Hand, amputation of, by machinery.. 561
Harrison on crystals on the peritoneum 180
Heart, Hallowell on rupture of the.. 152
Heart, cases of rupture of the ...... 154
Heart, lesions of the .............. 446
Heart, cartilaginous transformation of 447
Heart, lesions of the ............... 565
Hemiplegia, remarkable case of ... 239
Henderson on substernal aneurisms.. 183
Hernia, statistics of .............. 457
Hernia, radical cure of ............ 500
Hernia, mortification of .......... 546
Herpes confertus, case of .......... 385
Herpes circinatus, case of .......... 386
History of renal disease.. ......... 23
Holbrook's case of neuralgia of uterus 354
Homœopathy and allopathy, Uwins on 405
Human race, on the varieties of the.. 485
Human embryo vomited by a child.. 527
Humerus, ununited fracture of the.. 431
Hydrocele, injections of gaseous chlorine in......................... 513
Hydrocephalus chronicus externus .. 168
Hydrometra, Coley on ............ 357
Hydrometra, description of ........ 357
Hydrometra, cases of............. 358
Hysterical paralysis .............. 242

I

Idiopathic erysipelas .............. 251
Immediate causes of death.......... 567
Impracticable labour, table of the frequency of .................... 51
Increased value of life in this country 174
India, new medical journal in ...... 238
Indian rheumatism, causes of ...... 345
Infanticide, Dr. Cummin on ........ 322
Infants, common causes of death in.. 527
Injection, minute ................ 415
Injection, fine.................... 416
Injection, coarse......... ........ 416
Injection, cold .................. 416
Injections of gaseous chlorine in hydrocele......................... 513
Ink-bag of the cephalopoda ........ 203
Intermittent fever ................ 131
Internal hæmorrhage............... 61
Irish unanimity .................. 465

J

Johnson, Dr. on the modus operandi of medicines .................. 145
Johnson's case of occlusion of cystic duct, &c........................ 155
Johnson's case of paralysis of the tongue, &c. ................ .. 156
Johnson, H.J. his case of disease of the kidney ....... ............... 193
Joints, diseases of the ............ 248
Judd on urethritis and syphilis ...... 370

K

Kirkbride's Report of surgical cases.. 560
Knowles, his case of deformity of pelvis 143
Knox, his Anotomist's Instructor.... 408

L

Labour, proper period of .......... 48
Laryngismus stridulus, Dr. Ley on .. 1
Laryngismus stridulus, described as asthma ........................ 2
Laryngismus stridulus, called catarrhus suffocans ................ 2
Laryngismus stridulus, formidable symptom of.................... 3
Laryng. strid. association with carpopedal spasm ................. 3
Laryngismus stridulus, properties of, 4
Laryng. strid. pathology of ........ 4
Laryng. strid. some cases of ........ 5
Laryng. strid. dependence of on glandular enlargement .............. 9
Laryng. strid. causes of............ 10
Laryng. strid. causes of paroxysm in, 10
Laryng. strid. etiology of .......... 10

Laryng. strid. pathology of ........ 11
Laryng. strid. treatment of ........ 17
Laryng. strid. treatment of paroxysms 20
Latham on clinical medicine........ 75
Le Gros Clark on the nervous system, 481
Lee's Celsus ........ ............. 444
Lesions in the serous membranes.... 565
Lesions of the heart ........... ... 565
Lesions of the lungs .............. 566
Lesions of the liver .... ......... 566
Lesions of abdominal viscera...... .. 567
Lesions of the brain .............. 567
Ley on laryngismus stridulus ...... 1
Ley's doctrines, answers to objections 13
Lichen venereus ................ 379
Lichen, case of, with sore, &c....... 380
Lichen, with pustules, &c. .......... 381
Lichen, with sore and bubo ........ 382
Life in this country, increased value of 174
Ligaments, compendium of the, by M'Nab.................. .... 206
Liston, Mr. sketch of............... 238
Lithotomy and lithotrity ...... .... 465
Lithotrity in children, utility of ..... 466
Lithotomy à deux temps .......... 476
Local atrophy, three forms of ...... 122
London Hospital, statistics of the.... 555
London University, Charter of...... 583
Lunatics, rapid recovery of ........ 259
Lungs, lesions of the . ............. 566

M

Maculæ venereæ.................. 398
Maladie, convulsive ............... 506
Malcolmson on rheumatism ........ 341
Males and females, comparative mortality of ..................... 172
Malignant and hæmorrhagic tumors, 262
Malposition of the uterus .......... 36
Malposition of the uterus, causes of.. 37
Maltreatment of a fractured leg ..... 181
Management of twin labours......... 68
Manuel de médecine opératoire ..... 208
Mayo's, Mr., Outlines of Human Pathology .................... .... 293
Medical clubs .................... 574
Medical statistics ................. 169
Medical doctrine in France, state of.. 223
Medical witnesses' Bill ............ 568
Medical witnesses, summoning of.... 568
Medical witnesses, fees to.......... 569
Medicines, modus operandi of ...... 145
Medullary sarcoma entering the vertebral canal ..................... 531
Medullary carcinoma .............. 544
Medullary carcinoma, cases of ...... 544
Memory, temporary loss of ......... 453
Mercurial tremor, case of ......... 230
Mercury in onychia maligna ....... 474
Mesenteric disease, by W. A. Green.. 283
Middlemore on pterygium.......... 449
Midwifery, Collins on ............. 38
Midwifery, Hamilton on ........... 38
Midwifery, Ashwell on ............. 38
Mode of applying the ligature for aneurism....................... 561
Modus operandi of medicines ....... 145
Monomania, observations on ....... 216
Monomania, case of ............... 218
Monomanie raissonante............ 220
Monomanie raissonante, cases of .... 220
Moore on puerperal fever .......... 478
Mortality in Philadelphia under the age of puberty................... 169
Mortality of doctors .............. 174
Mortification of hernia ............ 546
Murray, Sir J. on the air-pump ..... 346
Mueller on erection of the penis ..... 188

N

Nævus maternus, preparation of .... 427
Natural labours .................. 44
Natural skeletons, on the making of.. 420
Nervous system, Dr. Hall on.... 92, 294
Nervous system, M. Le Gros Clark on 481
Neuralgia of the uterus, case of...... 354
Neville on carb. of soda in large doses, 150
New remedies, advantages of ....... 158
New Farthing Club ............... 571
New and nutritious aliment ........ 587
New Poor-law Act, report on ....... 325
Nieuwenhuys on autumnal fevers, &c. 131
Norris' case of fungoid tumor ...... 369
Nutrition of plants....... ......... 101
Nutrition of animals ....... ...... 102

O

Observations on monomania........ 215
Obstetric medicine, Dr. Davis on.... 36
Occlusion of cystic duct, Dr. Johnson's case of........ ................ 155
O'Meara, death of Mr. ............ 286
Operation for varicocele, Breschet's .. 463
Operation for stone in the bladder, mode of .. .................... 539
Operation for stone in the bladder, dangers of .................... 540
Operation for stone in the bladder, difficulties of ................. 542
Orders, the 7, of venereal eruptions.. 373
Ossification, on the treatment of .... 527
Osteology of the cranium and chest.. 490
Osteo-malacia, remarks on ......... 496
Osteo-malacia, case of ............ 497
Osteo-malacia in adults, remarks on.. 497
Osteo-malacia, symptoms of ....... 498
Osteo-malacia, local origin of ....... 498
Outlines of Human Pathology, Mr. Mayo's .......... ............ 293
Ovarian enlargement, treatment of .. 42
Ovarian tumors, Dr. Barlow on ..... 360
Ovarian tumor, fatal case of........ 361
Ovarian tumor simulating ascites, case 361
Ovarian tumor, antiphlogistic treatment of ..... ................ 363
Ovary, enlargement of ............ 41
Ozœna, cure of an ................ 514

P

Palmer's Dictionary of Terms ...... 117
Papulæ ........................ 377
Papulæ elongatæ ................. 378
Papulæ elongatæ, cases of ......... 379
Paracentesis abdominis in dropsical persons ...................... 445
Paralysis of the tongue, &c. Dr. Johnson's case of ........ .......... 156
Paralysis crescens ab imo .......... 169
Paris, prostitution of............... 333
Parkin on epidemic cholera ........ 480
Parson's report of out-patients...... 525
Partial convulsions, cases of......... 502
Patella, cases of fracture of the...... 561
Pathology of laryngismus stridulus .. 4
Pathology puzzled ................. 443
Pathology, contributions to ........ 446
Patronage of quacks ............... 576
Penis, Mueller on erection of the.... 188
Pennsylvania hospital .............. 560
Pericardium, ossification of ........ 447
Periostitis of the orbit, case of...... 176
Philosophy of decapitation ......... 151
Phlebitis, Mr. Douglas on .......... 263
Phlebitis, case of .................. 264
Phlebitis ......................... 533
Phthisis of 18 months standing, case of 562
Phthisis of 8 months standing, case of 564
Phymatosis ovata ................. 399
Phymatosis annulata ............... 400
Phymatosis verrucosa .............. 400
Physiological effects of creosote ..... 70
Pin, extraction of a, from the female bladder ...................... 452
Pins, swallowing of ................ 260
Placenta, retention of the.......... 60
Plants, nutrition of................ 101
Pneumothorax, Gerhard's reports on, 562
Poisoning from a viper-bite ....... 509
Poisoning by cantharides ........... 461
Polypus uteri within the womb...... 162
Porrigo lupinosa................... 241
Pregnancy, signs and duration of .... 42
Preparation of diseased bones ...... 424
Presentations with face to the pubes, 51
Presentations of the shoulder ..... 53
Preservation by drying ............ 410
Progressive development in the higher animals ...................... 489
Prolapsus of the uterus, Hamilton on, 40
Prolapsus of the umbilical cord...... 66
Prone position, Mr. Verral on the ... 275
Proposed modus operandi of medicines 148
Propriety of turning the child ...... 53
Prostitution of Paris, M. Duchatelet on 333
Prout on chemistry, meteorology, &c. 95
Provincial Association, Transactions of the ...................... 130, 347
Provincial Med. and Surg. Association 570
Psoriasis venerea ..... ........... 401
Psoriasis palmaria ................ 402
Pterygium, Mr. Middlemore on .... 449
Puerperal convulsions ............ 62
Puerperal convulsions, treatment of.. 63
Puerperal convulsions, case of ...... 63
Puerperal fever .................. 68
Puerperal fever, treatment of ....... 69
Puerperal fever, Dr. Alexander on ... 72
Puerperal fever, Dr. Alexander's treatment of ....................... 73
Puerperal fever, Mr. Moore on ...... 478
Punicious patch .................. 376

Q.

Quackery, its danger, &c. .......... 149
Quacks, patronage of.............. 576
Quain's anatomical plates.......... 207
Quomodo aqua hydropicis emittatur, 445

R.

Radical cure of hernia ............ 500
Railroads, tunnels on.............. 577
Rami nutritii arteriæ profundæ penis, 188
Rarefaction, cases of employment of.. 347
Recovery from dislocation of spine, &c. 185
Remarks on osteo-malacia.......... 496
Remarks on form of partial convulsions, 501
Renal disease, Dr. Bright on........ 23
Renal disease, hæmaturia a precursor of 23
Renal disease, history of ......... 23
Renal disease, cases of ........... 24
Renal disease, treatment of ........ 31
Renal disease, good effects of purgatives in ...................... 33
Reparation of fracture, stages of .... 435
Report of the Birmingham Infirmary, 528
Report of surgical cases, by Kirkbride, 560
Report on the New Poor-Law Act... 325
Reporting from medical societies .... 279
Respiration, chemical theory of .... 112
Retention of the placenta .......... 60
Retention of urine, use of catheter in, 261
Rheumatism, Bouillaud's treatment of, 90
Rheumatism, Mr. Malcolmson on.... 341
Rheumatism, parts affected in ...... 341
Rigby on philosophy of decapitation.. 151
Roots on chronic laryngitis ........ 449
Roots on acute pericarditis ........ 455
Rupia venerea.................... 387
Rupia simplex.................... 388
Rupia, case of.................... 390
Rupture of the uterus or vagina .... 64
Rupture, more prevalent in males or females? ...................... 458
Ryan's Formulary of Hospitals...... 199
Ryan's Formulary of Hospitals, inaccuracies of .................... 200
Ryan's Dr., reclamation .......... 581
Ryland's report of out-patients...... 532

S.

St. Thomas's Hospital ............ 534
Scarlet fever..................... 133
Scarlet fever, case of.......... .... 134
Scrofula ......... ............ 20
Secale cornutum, Mr. Chavasse on .. 349

Secale cornutum, efficacy of.... ... 351
Secondary symptoms .............. 396
Secondary inflammations .......... 266
Secondary abscesses, pus the cause of, 268
Serous membranes, lesions in the.... 565
Serres on progressive development in the higher animals.......... ... 489
Shoulder, presentations of the ...... 53
Siamese Twins .................... 493
Siamese Twins, nature of the cord which unites the................ 494
Signs and duration of pregnancy .... 42
Skeleton ......................... 418
Skeleton, position of the .......... 422
Skeleton of fishes, preservation of the, 423
Skull, compound fracture of the .... 167
Small-pox ........................ 135
Small-pox at Vienna in 1835 ....... 500
Smith's contributions to pathology .. 446
Soda, Neville on carb. of, in large doses, 150
Soda, beneficial effects of, in children, 151
South's St. Thomas's hospital reports, 534
Spider, severe symptoms from bite of, 508
Spili ciccinii ..................... 398
Spili cruentati.................... 398
Spili cuprei ...................... 399
Spontaneous combustion .......... 577
Spurzheim, skull of ............. 448
Squamæ venereæ .............. ... 401
Stammering, Mr. Cull on .......... 319
State of medical doctrine in France .. 223
Statistics of factories .............. 175
Statistics of fever ............... 547
Statistics of the London Hospital.... 555
Sternutation, extraordinary cure of .. 501
Stomach, general operations of the .. 106
Stomach, reducing power of the ... 107
Stone in the bladder .............. 537
Strangulated umbilical hernia, excision of the cyst of a ............ 182
Substernal aneurisms, Dr. Henderson on ......................... 183
Substernal aneurism, signs of .. ... 183
Substernal aneurism, sounds of ..... 184
Successful amputation during traumatic gangrene ................ 187
Surgeon's practical guide, Cutler's .. 206
Surgical cases, report of, by Kirkbride, 560

T.

Tables of the numbers admitted, &c. in the London Hospital in 1828-33 .. 558
Temporary loss of memory.......... 453
Testis, liability to inflammation of the, 395
Tetanus, use of colchicum in ....... 161
Tight-lacing, Coulson on .......... 460
Tooth-ache, remedy for ............ 71
Treatise on the properties of creosote, 69
Treatment of laryngismus stridulus .. 17
Treatment of renal disease ......... 31
Treatment in uterine hæmorrhage ... 59
Treatment of puerperal convulsions .. 63
Treatment of puerperal fever........ 69
Treatment of vermination........ ... 74
Treatment of ossification .......... 527
Tunnels in railroads .............. 577
Turning the child, propriety of... .. 53
Turpentine, use of, in museums .... 414
Twin-labours, management of ...... 68
Twins, case of.................... 495

U. & V.

Umbilical cord, prolapsus of the .... 66
Unfermented bread..... .......... 586
Ununited fracture, five cases of...... 429
Ure on the chloride of zinc.......... 190
Urethritis and syphilis, Mr. Judd on.. 370
Urine, detection of sulphate of quinine in ......................... 215
Use of the cochlea ................ 177
Use of catheter in retention of urine.. 261
Uterine hæmorrhage .............. 56
Uterine hæmorrhage, case of recovery from ........................ 57
Uterine hæmorrhage, treatment in ... 59
Utero, case of crying of the child in.. 66
Uterus, malposition of ........... 36
Uterus, dilapsion of .............. 36
Uterus, prolapsion of.............. 36
Uterus, procidentia of............. 36
Uterus, treatment of descents of the.. 37
Uterus, Dr. Hamilton on prolapsus of, 40
Uterus, rupture of the ............ 64
Uwins on homoeopathy and allopathy, 405
Vagina, rupture of the ............ 64
Vagina, congenital occlusion of the .. 522
Vaginal hernia of the bladder ....... 164
Varieties of the human race ........ 485
Varnish, use of, in museums ....... 411
Vegetable substances .............. 95
Venereal eruptions................ 371
Venereal eruptions, seven orders of .. 373
Venereal exanthemata ......... .. 375
Venereal pustules ................ 391
Venereal tubercles .......... ..... 399
Ventral hernia ................... 531
Vermination ..................... 74
Vermination, treatment of ...... .... 74
Verral on the prone position........ 275
Vesiculæ venereæ ................ 383
Vesiculæ venereæ, cases of ......... 383
Viper-bite, poisoning from a........ 509

W.

Want of the sagittal suture, Mr. Wallace's cases of ................ 285
Wet preparations, putting up of .... 413

